# Influence of Pre-Freezing Temperature on the Corneal Endothelial Cytocompatibility and Cell Delivery Performance of Porous Hyaluronic Acid Hydrogel Carriers

**DOI:** 10.3390/ijms160818796

**Published:** 2015-08-11

**Authors:** Jui-Yang Lai

**Affiliations:** 1 Institute of Biochemical and Biomedical Engineering, Chang Gung University, Taoyuan 33302, Taiwan; E-Mail: jylai@mail.cgu.edu.tw; Tel.: +886-3-211-8800 (ext. 3598); Fax: +886-3-211-8668; 2 Biomedical Engineering Research Center, Chang Gung University, Taoyuan 33302, Taiwan; 3 Molecular Medicine Research Center, Chang Gung University, Taoyuan 33302, Taiwan; 4 Center for Tissue Engineering, Chang Gung Memorial Hospital, Taoyuan 33305, Taiwan

**Keywords:** hyaluronic acid, pre-freezing temperature, porous hydrogel carrier, corneal endothelial cytocompatibility, cell delivery performance

## Abstract

The development of porous hyaluronic acid (HA) hydrogels for corneal endothelial tissue engineering is attractive because they can be used as functional cell delivery carriers to help in the reconstruction of damaged areas. The purpose of this study was to investigate the corneal endothelial cytocompatibility and cell delivery performance of porous HA hydrogel biomaterials fabricated at different pre-freezing temperatures. As compared to their counterparts prepared at −80 °C, the HA samples fabricated at higher pre-freezing temperature (*i.e.*, 0 °C) exhibited a larger pore size and higher porosity, thereby leading to lower resistance to glucose permeation. Live/dead assays and gene expression analyses showed that the restricted porous structure of HA carriers decreases the viability and ionic pump function of cultured corneal endothelial cells (CECs). The results also indicated that the porous hydrogel biomaterials fabricated at high pre-freezing temperature seem to be more compatible with rabbit CECs. In an animal model of corneal endothelial dysfunction, the wounded rabbit corneas receiving bioengineered CEC sheets and restricted porous-structured HA carriers demonstrated poor tissue reconstruction. The therapeutic efficacy of cell sheet transplants can be improved by using carrier materials prepared at high pre-freezing temperature. Our findings suggest that the cryogenic operation temperature-mediated pore microstructure of HA carriers plays an important role in corneal endothelial cytocompatibility and cell delivery performance.

## 1. Introduction

Biomaterial hydrogels can provide a favorable microenvironment for cell organization and tissue regeneration [1]. Therefore, the tissue-compatible hydrogel carriers are widespread in current cell delivery applications. To improve chronic wounds via differentiation and paracrine effects, Hassan *et al.* have reported a strategy for the delivery of adipose-derived stem cells by using *in situ* cross-linked hybrid hydrogel composed of poly(ethylene glycol) (PEG)-based hyperbranched copolymer and hyaluronic acid (HA) [2]. For nucleus pulposus cell delivery to the region of the pathological intervertebral disc, Francisco *et al.* have shown that the incorporation of laminin ligands into an injectable PEG hydrogel carrier is beneficial to promote the survival and phenotype of transplanted cells [3]. Liu *et al.* have also demonstrated that the constructs fabricated by culturing human umbilical cord mesenchymal stem cells in a fibrin hydrogel containing degradable microbeads display enhanced cell viability and successful myogenic differentiation with the formation of multinucleated myotubes [4]. These studies reflect the importance of using natural or synthetic biomaterials as functional cell delivery carriers.

Due to its inherent biocompatibility, HA is one of the most commonly-used ophthalmic biomaterials in clinical practice. This polysaccharide has been applied as a viscoelastic agent for cataract surgery [5] and deep lamellar keratoplasty [6]. Our group has previously investigated the fabrication of bioengineered corneal keratocyte spheroids on HA coatings and found that the molecular weight of HA is crucial to determine the cell–matrix and cell–cell interactions [7]. Oxidized HA-modified biopolymer microcarriers can effectively promote corneal keratocyte growth *in vitro* [8]. In addition, to serve as a corneal endothelial cell (CEC) sheet carrier for intraocular delivery, the HA disc should be chemically cross-linked to overcome the rapid dissolution of polysaccharide molecule in aqueous environments [9]. The *in vivo* biocompatibility studies show that the continued residence of glutaraldehyde-treated HA implants elicits severe ocular tissue responses [10]. However, no adverse foreign body reaction is noted for the rabbits receiving carbodiimide-modified HA materials. The cross-linking degree of hydrogel biomaterials can be further optimized by tuning the solvent composition for carbodiimide treatment of HA molecules [11].

To improve the aqueous humor circulation, it is highly desirable to develop porous hydrogel carriers for intraocular delivery of CEC sheets [12]. Therefore, in this work, the porous HA carriers were prepared by using a technique based on simple stirring operation and freeze-drying. Since the processing method of porous hydrogel biomaterials does not involve any toxic porogen agents, the safety risk can be tremendously diminished. Given that pre-freezing temperature is one of the most important factors affecting the size of ice crystals during cryogenic formation, numerous investigators have performed the characterization studies on this subject. The pre-freezing temperature-mediated pore microstructure of collagen/HA [13], collagen [14], gelatin-siloxane [15], gelatin [16], poly(l-glutamic acid) [17], alginate/galactosylated chitosan [18], starch/chitosan [19], chitosan/galactosylated HA [20], chitosan [21], poly(l-lactide)/chitosan [22] and hydroxyapatite-chitosan-gelatin [23] scaffold materials has been reported in the literature. However, to the best of our knowledge, the application of freeze-dried HA hydrogel carriers for CEC sheet delivery has not been evaluated.

The purpose of this study was to determine the corneal endothelial cytocompatibility and cell delivery performance of porous HA hydrogel carriers fabricated at different pre-freezing temperatures (*i.e.*, high pre-freezing temperature (HFT, 0 °C) and low pre-freezing temperature (LFT, −80 °C)). The variation of porous microstructure of HA samples was assessed by scanning electron microscopy (SEM) observations and porosity measurements. The glucose permeability of delivery carriers was also detected to determine the resistance to nutrient transport. Live/dead bioassays were used to measure the *in vitro* biocompatibility of HA carriers. The ionic pump function of CECs was examined by analyzing the Na^+^,K^+^-ATPase alpha 1 subunit (ATP1A1) gene expressions. On the other hand, to evaluate the cell delivery performance of carrier materials, an animal model of corneal endothelial dysfunction was employed. After intraocular delivery of HA-CEC sheet constructs to the rabbit cornea denuded of endothelium, the slit-lamp biomicroscopy and specular microscopy were conducted to observe changes in corneal tissue morphology and structure.

## 2. Results and Discussion

### 2.1. Characterization of Porous Structure

**Figure 1:**
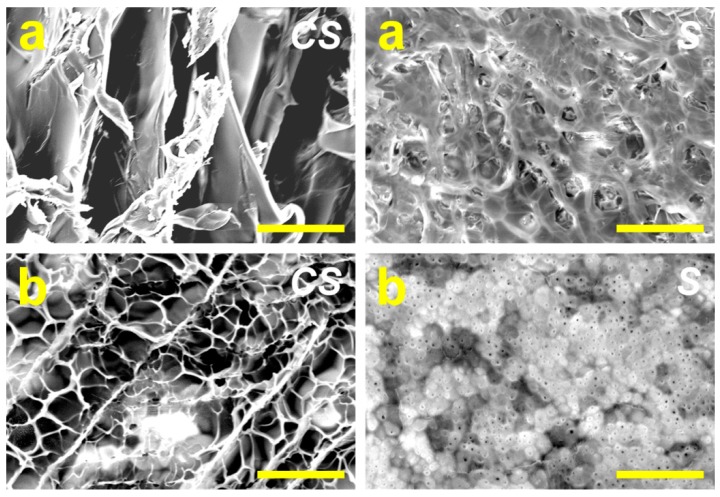
Scanning electron microscopic images of various hyaluronic acid (HA) carriers. (**a**) High pre-freezing temperature (HFT) and (**b**) low pre-freezing temperature (LFT) groups. CS: cross-section; S: surface. Scale bars: 200 μm.

SEM is a frequently-used imaging technique to examine morphological changes in materials science and engineering research. Figure 1 shows the cross-sectional and surface images of HA carriers fabricated at different pre-freezing temperatures. Although the carriers from both groups showed porous three-dimensional microstructure, the distinct pore morphologies were observed in the SEM. The HFT samples contained larger pores in the interior of the HA carriers than their LFT counterparts. It has been documented that the variations in the pore microstructure of biomaterial hydrogels reflect the importance of heat transfer rates during the cryogenic treatment [24]. At lower freezing temperature, the formation of many nuclei of ice crystals may lead to the generation of small-sized pores in the hydrogel scaffolds. The differences in the nucleation and growth of ice crystals due to cryogenic operation conditions, such as freezing temperature, were also noted in the surface morphology between HFT and LFT groups. While a few small pores were present on the surface of HA carriers prepared by freezing at 0 °C, the samples frozen at −80 °C showed fine-grained microstructures with almost no pores. The findings support earlier research, demonstrating that freeze-dried porous biomaterials may simultaneously exhibit a dense surface skin layer [16].

**Figure 2:**
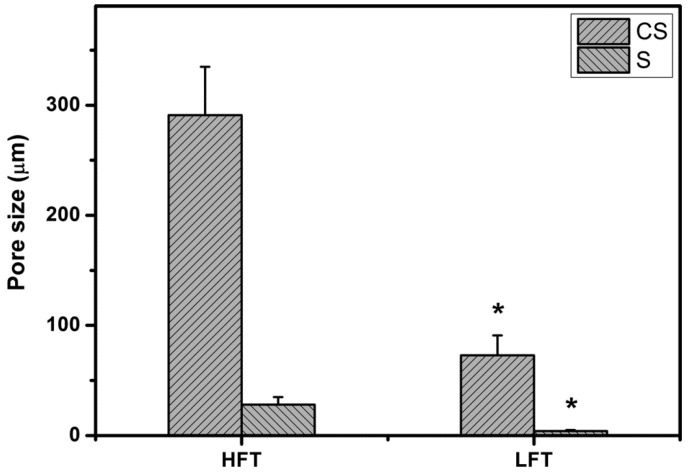
Pore size of various HA carriers. An asterisk indicates statistically-significant differences (* *p* < 0.05; *n* = 4) *vs.* HFT (compared only within the CS or S groups).

**Figure 3:**
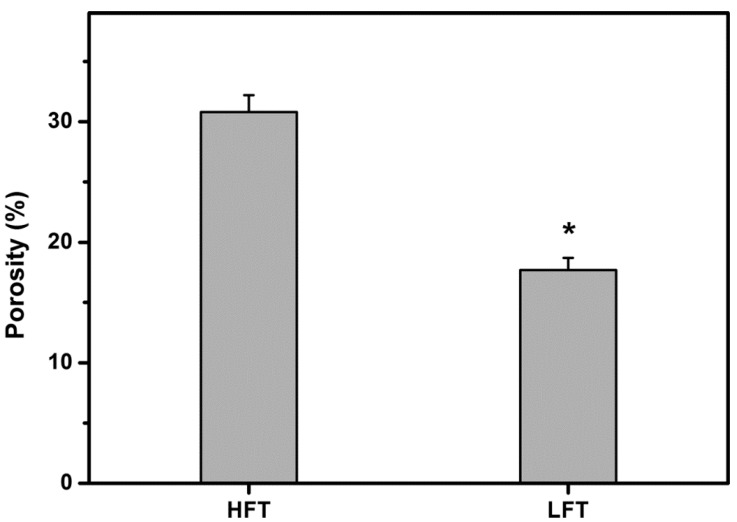
Porosity of various HA carriers. An asterisk indicates statistically-significant differences (* *p* < 0.05; *n* = 4) as compared to the HFT groups.

Figure 2 shows the pore size of HA carriers fabricated at different pre-freezing temperatures. The cross-sectional pore size significantly differed between HFT (291 ± 44 μm) and LFT (73 ± 18 μm) groups (*p* < 0.05). Additionally, in the HFT groups, significantly larger surface pore size (28 ± 7 μm) was observed than in the LFT (4 ± 1 μm) groups (*p* < 0.05), demonstrating a similar trend. Quantification from SEM images depicts that with decreasing cryogenic operation temperature from 0 to −80 °C, the pore size of freeze-dried HA hydrogels is reduced due to enhanced nucleation and formation of small-sized ice crystals. Figure 3 shows the porosity of HA carriers fabricated at different pre-freezing temperatures. The material samples from HFT groups had a porosity of 30.8% ± 1.4%. This was significantly higher than those of the LFT groups (*p* < 0.05). The results indicate that the growth of large-sized ice crystals at higher freezing temperature also exerts a profound influence on the creation of more pore space in the fabricated HA hydrogels. Park *et al.* have previously characterized the porous structure of collagen/HA scaffolds prepared at −70 °C and found that the samples have an average porosity of around 60% [13]. In this study, the HA carriers fabricated at −80 °C exhibit a porosity of 17.7% ± 1.0%, which is markedly lower than the corresponding value reported in the case of the freeze-dried collagen/HA scaffold system. These findings suggest that the addition of another component (*i.e.*, collagen) to the HA samples may alter the pore volume of biopolymer carriers during the cryogenic treatment.

### 2.2. Glucose Permeation Studies

**Figure 4:**
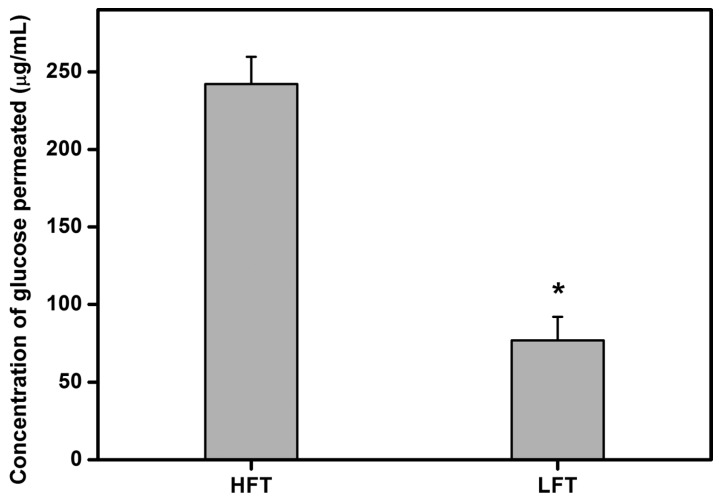
Concentration of glucose permeated through various HA carriers at 34 °C. An asterisk indicates statistically-significant differences (* *p* < 0.05; *n* = 6) as compared to the HFT groups.

Since the cell carrier implants possibly restrict intraocular nutrient transport, it is necessary to determine the nutrient permeability of the hydrogel materials. In this study, the amounts of glucose permeated through various HA hydrogel samples at 34 °C were quantitated using a glucose assay kit (Figure 4). The glucose concentration in the HFT groups was 242.1 ± 17.6 μg/mL. It was significantly higher than those in the LFT (76.8 ± 15.3 μg/mL) groups (*p* < 0.05), indicating that the glucose permeability of porous hydrogel carriers may depend on the pre-freezing temperature for the HA solution. These findings also imply that the solute permeation can be modulated with the variation of porous microstructure because the pore size and porosity of biomaterials are highly correlated with cryogenic operation conditions, such as freezing temperature. Although the cross-sectional pore dimensions are an important feature in glucose permeation through the HA hydrogels, the surface pore dimensions seem to be critical for the outcome. It has been reported that the formation of surface pores on the microcapsules may allow the bidirectional diffusion of oxygen and nutrients, indicating the role of surface pores in mass transport [25]. While a few small pores are present on the carrier surface of HFT samples, the LFT materials shows fine-grained microstructures with almost no surface pores. In addition to pore size, the pore wall thickness is another key structural parameter affected by the freezing temperature. Mao *et al.* have previously investigated the effect of pre-freezing temperature on the microstructure of bilayer chitosan-gelatin scaffolds and found that the decrease in freezing temperature during the processing of biopolymer matrix composite materials may lead to thinner pore wall thickness [26]. Theoretically, the glucose diffusion rate increases with reducing pore wall thickness. The HA carriers fabricated at lower pre-freezing temperature, however, significantly inhibited the glucose permeation. In contrast, the hydrogel samples with a larger pore size exhibit lower resistance to nutrient transport, suggesting that the pore size and porosity play dominant roles in glucose permeability of cell delivery carriers.

###  2.3. In Vitro Biocompatibility Studies

**Figure 5:**
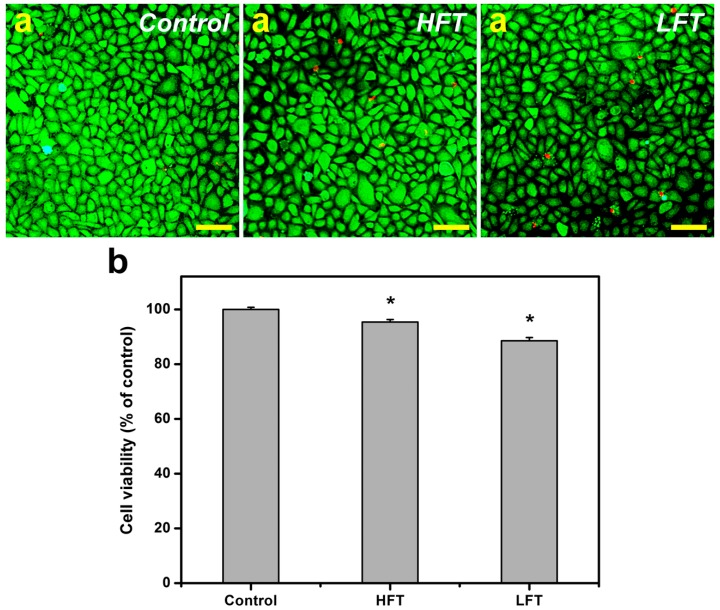
Cell viability of rabbit corneal endothelial cell (CEC) cultures was determined by staining with the Live/Dead Viability/Cytotoxicity Kit in which the live cells fluoresce green and dead cells fluoresce red. (**a**) Fluorescence images of cells in controls (without test materials) after 8 h of direct contact with different types of HA samples. Scale bars: 50 μm; (**b**) Quantitative results are expressed as the percentage of control groups (viability of cells cultured in the absence of test materials). An asterisk indicates statistically-significant differences (* *p* < 0.05; *n* = 3) as compared to the control groups.

Here, using a primary rabbit CEC culture system, we investigated the *in vitro* biocompatibility of porous HA carriers for bioengineered cell sheet delivery. The results of live/dead staining provide insights into the viability of CEC cultures exposed to various HA samples. Figure 5a depicts that the majority of cells from the control groups were alive. After 8 h of direct contact with the HFT carrier materials, the rabbit CECs emitted prominent green fluorescence, which is a pattern indicative of large amounts of live cells. In addition, a few cells emitting red fluorescence were identified as dead cells. Although the same number of red-stained nuclei was noted, the number of green fluorescent live cells was reduced in the LFT groups. The viability level in the control, HFT and LFT groups was 100.0% ± 0.8%, 95.4% ± 0.9% and 88.5% ± 1.2%, respectively (Figure 5b). The values showed significant differences between these three groups (*p* < 0.05), suggesting that the variations in cell viability are sensitive to the alterations in the pore microstructure of HA hydrogels fabricated at different pre-freezing temperatures. The mass transport limitations associated with the low porosity of synthetic porous biomaterials are found to be important for cell survival [27]. One possible explanation for our results may be that insufficient glucose transport into the cultured CECs contributes to the decreased cell survival upon exposure to the porous HA carriers with relatively low porosities. A similar phenomenon has also been reported in the case of rabbit CECs treated with porous gelatin hydrogels that have a porosity of less than 35% [28].

**Figure 6:**
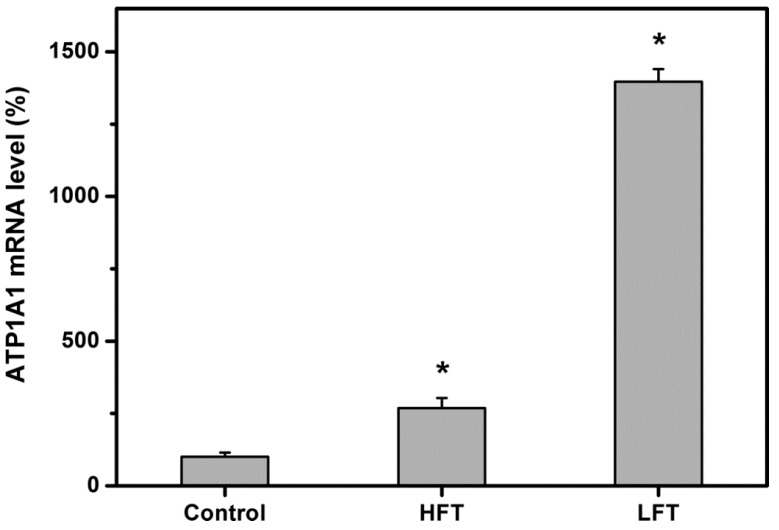
Gene expression level of ATP1A1 in rabbit CECs after 8 h of direct contact with various HA carriers, measured by real-time reverse transcription polymerase chain reaction. Normalization was done by using GAPDH. Data in the experimental groups are percentages relative to that of control groups (cells cultured in the absence of HA materials). An asterisk indicates statistically-significant differences (* *p* < 0.05; *n* = 4) as compared to the control groups.

*In vivo*, the corneal endothelium acts as a physiological barrier to maintain tissue homeostasis and to regulate tissue clarity [29]. To further understand the physiological function of delivered CECs affected by the porous microstructure of hydrogel carriers, the quantitative real-time RT-PCR was performed to detect the gene expression profiles. Figure 6 shows the gene expression of membrane transport protein (*i.e.*, ATP1A1) in the cultured rabbit CECs 8 h after direct contact with the tested materials. The measured expression level in the control cultures was defined as 100%. Our data demonstrated that in the presence of HA hydrogels, the CEC cultures had significantly higher ATP1A1 mRNA levels than did those of the control groups (*p* < 0.05). Additionally, in the LFT groups, the ATP1A1 gene expression was 1396.5% ± 44.0%, which was significantly higher than those of the HFT (268.3% ± 35.1%) groups (*p* < 0.05). The variations in the altered expression level of ATP1A1 reflect the importance of cryogenic operation temperature-mediated pore microstructure of carrier materials in changing CEC ionic pump function. As mentioned earlier, the HA carriers fabricated at −80 °C have almost no surface pores, thereby leading to abnormal transmembrane transport. However, even the existence of a few pores on the surface of HA samples prepared by freezing at higher temperature, the limited pore size and porosity do not suffice to prevent upregulation of ATP1A1 mRNA expression. In terms of *in vitro* biocompatibility, the LFT carrier material seems not to be well tolerated by the cultured CECs, since its restricted pore structure may contribute to abnormal cell function and loss of viability.

###  2.4. In Vivo Transplantation Studies

**Figure 7:**
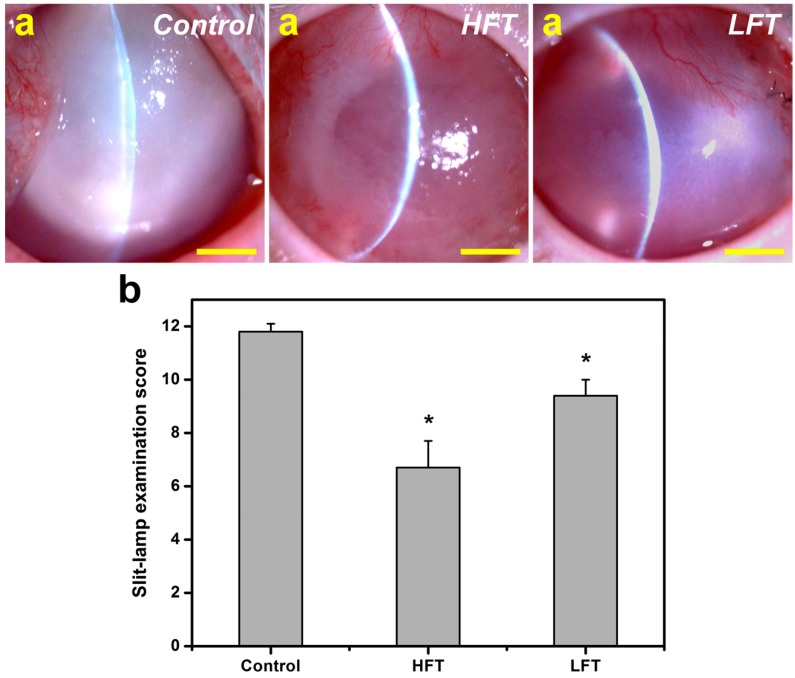
(**a**) Representative slit-lamp biomicroscopic images of rabbit eyes four weeks after surgical treatment of corneal endothelial dysfunction. Control group: cornea denuded of endothelium; HFT group: endothelial scrape-wounded cornea implanted with HFT carriers and bioengineered CEC sheet; LFT group: endothelial scrape-wounded cornea implanted with LFT carriers and bioengineered CEC sheet. Scale bars: 5 mm; (**b**) Slit-lamp examination scores of rabbit eyes four weeks after surgical treatment of corneal endothelial dysfunction. An asterisk indicates statistically-significant differences (* *p* < 0.05; *n* = 6) as compared to the control groups.

To investigate the application potential of porous cell sheet carriers in corneal endothelial regenerative medicine, an *in vivo* study was performed with an animal model of corneal endothelial dysfunction. As described in our previous report [30], the cell/biopolymer construct implants can be fabricated on thermo-responsive culture substrates and delivered to intraocular tissues through a minimally-invasive approach. Ophthalmic evaluations were made in animal eyes four weeks after surgical treatment of corneal endothelial dysfunction. Representative slit-lamp biomicroscopic images for each group are shown in Figure 7a. In the control groups, severe corneal opacity and edema were observed in the rabbits denuded of corneal endothelium, indicating successful establishment of the animal model of corneal endothelial dysfunction. While the endothelial scrape-wounded corneas implanted with HFT carriers and bioengineered CEC sheet grafts showed improved tissue clarity, their traumatized counterparts receiving LFT carriers and bioengineered CEC sheet grafts remained cloudy. In particular, in the LFT groups, corneal neovascularization became more developed than those of the HFT groups. The slit-lamp examination score for each group is shown in Figure 7b. Before surgery, the overall ocular scores were close to zero. In the control groups, the total score was 11.8 ± 0.3, indicating severe corneal tissue damage after mechanical removal of endothelium. By contrast, the implantation of HA-CEC sheet constructs in the endothelial scrape-wounded cornea led to a significantly lower score (*p* < 0.05). The animals in the HFT groups had a score of 6.7 ± 1.0, which was significantly lower than that of the LFT (9.4 ± 0.6) groups (*p* < 0.05). It has been documented that the pore microstructure of cell sheet delivery carriers is involved in the maintenance of nutrition and the avascularity of the normal cornea [12]. For the first time, here, we demonstrated that the cryogenic operation temperature-mediated pore microstructure of carrier materials significantly affects the therapeutic efficacy of cell sheet grafts used to treat corneal endothelial dysfunction.

**Figure 8:**
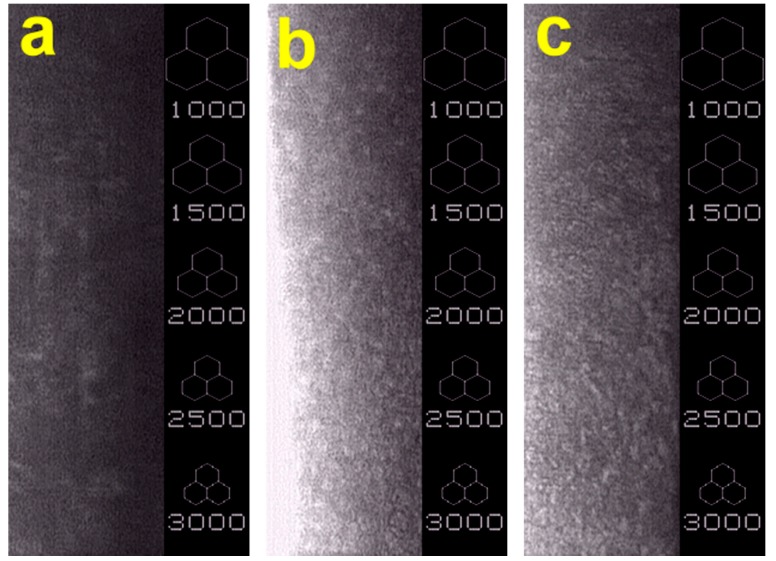
Representative specular microscopic images of rabbit eyes four weeks after surgical treatment of corneal endothelial dysfunction. (**a**) Control group: cornea denuded of endothelium; (**b**) HFT group: endothelial scrape-wounded cornea implanted with HFT carriers and bioengineered CEC sheet; and (**c**) LFT group: endothelial scrape-wounded cornea implanted with LFT carriers and bioengineered CEC sheet.

Reconstruction of the corneal endothelium using bioengineered cell sheets and porous HA carriers was further examined by specular microscopy, which is a non-invasive photographic technique to analyze the endothelium monolayer at the cellular level [31]. Representative specular microscopic images for each group are shown in Figure 8. In the control groups, almost no CECs were identified in the corneal tissue at four weeks post-surgery, indicating complete mechanical stripping of native corneal endothelium from Descemet’s membrane. Results of quantitative specular microscopic analysis of rabbit corneal endothelium demonstrated that the cell density is close to zero. The variations in corneal endothelial cell morphology were noted for the wounded corneas receiving bioengineered CEC sheet grafts and different porous HA carriers. In the HFT groups, the implanted cell sheet was well attached to the posterior surface of cornea. Only a few cells were damaged due to the insufficient pore size and porosity of carrier materials. The mean endothelial cell count was 2351 ± 167 cells/mm^2^. By contrast, in the LFT groups, the relatively restricted porous structure of delivery carriers resulted in a clearly evident cell injury. The animals had a significantly lower CEC density (1286 ± 110 cells/mm^2^) compared to the HFT groups (*p* < 0.05). Additionally, the remaining CECs on Descemet’s membrane did not exhibit a typical hexagonal shape. These findings suggest that the hydrogel samples fabricated at −80 °C may be unsuitable for use as cell sheet carriers in the treatment of corneal endothelial dysfunction.

## 3. Experimental Section

### 3.1. Materials

Hyaluronic acid sodium salt was obtained from Kewpie (Tokyo, Japan) as a dry powder. It was made by the fermentation method and was highly purified. According to information from the supplier, the HA samples used in this study had a weight-average molecular weight of around 1100 kDa. 1-ethyl-3-(3-dimethyl aminopropyl) carbodiimide hydrochloride, glucose and glucose assay kit (glucose oxidase/peroxidase reagent and o-dianisidine reagent) were purchased from Sigma-Aldrich (St. Louis, MO, USA). Balanced salt solution (BSS, pH 7.4) was obtained from Alcon Laboratories (Fort Worth, TX, USA). Phosphate-buffered saline (PBS, pH 7.4) was purchased from Biochrom AG (Berlin, Germany). Medium 199, gentamicin, Hanks’ balanced salt solution (HBSS, pH 7.4), trypsin-ethylenediaminetetraacetic acid (EDTA) and TRIzol reagent were purchased from Gibco-BRL (Grand Island, NY, USA). Collagenase type II was purchased from Worthington Biochemical (Lakewood, NJ, USA). Fetal bovine serum (FBS) and the antibiotic/antimycotic (A/A) solution (10,000 U/mL of penicillin, 10 mg/mL of streptomycin and 25 μg/mL of amphotericin B) were obtained from Biological Industries (Kibbutz Beit Haemek, Israel). All other chemicals were of reagent grade and used as received without further purification.

### 3.2. Preparation of Porous HA Carriers

The aqueous HA solutions of 0.5 wt % were prepared by dissolution of HA powder in double-distilled water. Before lyophilization at −55 °C for 2 days, the aqueous solution was cooled to 25 °C, stirred with a rate of 350 rpm for 20 min and frozen at 0 °C (HFT group) or −80 °C (LFT group) for 24 h to form ice crystals.

All fabricated HA hydrogel sheets were further cross-linked by immersing in an acetone/water mixture (85:15, *v*/*v*, pH 4.75) of 10 mM 1-ethyl-3-(3-dimethyl aminopropyl) carbodiimide (EDC) [11]. The cross-linking reaction was allowed to proceed at 25 °C for 2 days. The samples were thoroughly washed with double-distilled water to remove excess EDC and urea by-product. Using a 7 mm-diameter corneal trephine device, the hydrogel sheets were cut out to create carrier discs (~700 μm in thickness). The water content of HA hydrogels was determined to evaluate their cross-linking degree as described previously [9]. In this study, the samples from HFT and LFT groups have similar water contents (83%–87%).

### 3.3. Characterization of Porous Structure

Specimens were prepared for scanning electron microscopy (SEM) as described previously [9]. Small pieces of the hydrogel discs were mounted onto stubs and gold coated in a sputter coater (Hitachi, Tokyo, Japan). The cross-section and surface morphologies of the HA carriers were examined using a Hitachi S-3000N SEM with an accelerating voltage of 10 kV. Twenty different pores were randomly selected, and the average pore diameters were calculated. Results were averaged on four independent runs.

The solvent replacement method was used for porosity measurements [32]. Each HA disc was first dried to constant weight (*W*_i_) *in vacuo*. The test samples were immersed in absolute ethanol overnight, blotted with tissue paper to remove excess ethanol on the surface and weighed (*W*_f_) immediately. The porosity (%) was calculated as ((*W*_f_ − *W*_i_)/*V*ρ) × 100, where *V* is the volume of the hydrogel disc and ρ is the density of absolute ethanol. Results were averaged on four independent runs.

### 3.4. Glucose Permeation Studies

Glucose permeation studies were performed at 34 °C using a horizontal glass diffusion cell (PermeGear, Hellertown, PA, USA) as described previously [12]. The HA samples were placed between the two chambers. The donor chamber was filled with a 6.9 μmol/mL (the glucose concentration of aqueous humor in rabbit) glucose solution in BSS (3 mL) and the receptor chamber with BSS (3 mL). During the measurements, all solutions were stirred continuously to reduce boundary layering of glucose. After 8 h, the receptor chamber was sampled and analyzed using a glucose assay kit following the manufacturer’s instructions. Photometric readings at 540 nm were measured with a spectrophotometer (Thermo Scientific, Waltham, MA, USA) and compared with a standard curve of known glucose concentrations. Results were averaged on six independent runs.

### 3.5. In Vitro Biocompatibility Studies

All animal procedures were approved by the Institutional Review Board and were performed in accordance with the Association for Research in Vision and Ophthalmology (ARVO) Statement for the Use of Animals in Ophthalmic and Vision Research. Sixteen adult New Zealand white rabbits (National Laboratory Animal Breeding and Research Center, Taipei, Taiwan) were used for *in vitro* biocompatibility studies. Primary rabbit CECs were prepared according to previously-published methods [9]. The Descemet’s membrane-corneal endothelium complex was digested using 2 mg/mL collagenase in HBSS for 1 h at 37 °C. Thereafter, the CECs were collected and resuspended in regular culture medium containing Medium 199 as a basal medium, 10% FBS, 50 μg/mL gentamicin and 1% A/A solution. Confluent monolayers were subcultured by treating with trypsin-EDTA for 2 min and seeded at a 1:4 split ratio. Only second-passage cells were used.

Rabbit CECs (7 × 10^4^ cells/well) were seeded in 24-well plates containing regular growth medium and incubated overnight to allow cell attachment. After 1 week of cultivation, the HA discs sterilized in a graded series of ethanol solutions were placed on the apical cell surface in direct contact with the confluent cultures. Rabbit CEC cultures without contacting disc samples served as control groups. Following incubation for 8 h, the cell viability was determined using the Live/Dead Viability/Cytotoxicity Kit from Molecular Probes (Eugene, OR, USA) as described previously [33]. Cells were observed and imaged under fluorescence microscopy (Axiovert 200M; Carl Zeiss, Oberkochen, Germany), and three different areas were randomly selected and counted at 100× to further quantify the average number of green fluorescent live cells. All experiments were performed in triplicate, and the results were expressed as relative cell viability when compared to control groups.

On the other hand, the gene expression levels were measured after 8 h of direct contact between the CEC cultures and sterilized HA carriers. The total RNA was isolated from cells with TRIzol reagent according to the manufacturer’s procedure [7]. Reverse transcription of the extracted RNA (1 μg) was performed using ImProm-II (Promega, Madison, WI, USA) and Oligo(dT)_15_ primers (Promega). The primers used to amplify the rabbit Na^+^,K^+^-ATPase alpha 1 subunit (ATP1A1) complementary DNA (cDNA) were 5′-GTCTTCCAGCAGGGCATGAA-3′ (sense) and 5′-TAAGGGCAACACCCATTCCA-3′ (antisense). The sequences of the primer pair used to amplify the internal control cDNA, glyceraldehyde-3-phosphate dehydrogenase (GAPDH), were 5′-TTGCCCTCAATGACCACTTTG-3′ (sense) and 5′-TTACTCCTTGGAGGCCATGTG-3′ (antisense). Quantitative real-time reverse transcription polymerase chain reaction (RT-PCR) was performed on a Light-Cycler instrument (Roche Diagnostics, Indianapolis, IN, USA) according to the manufacturer’s instructions with FastStart DNA Master SYBR Green I reagent (Roche Diagnostics). Each sample was determined in quadruplicate, and the gene expression results were normalized to the level of GAPDH mRNA.

### 3.6. In Vivo Transplantation Studies

Eighteen adult New Zealand white rabbits (National Laboratory Animal Breeding and Research Center) weighing 3.0–3.5 kg and the HA hydrogel carriers sterilized in a graded series of ethanol solutions were used for *in vivo* transplantation studies. All animal procedures were approved by the Institutional Review Board and were carried out in accordance with the ARVO Statement for the Use of Animals in Ophthalmic and Vision Research. Bioengineered CEC sheets were harvested from the thermo-responsive culture surfaces by reducing the incubation temperature from 37 down to 20 °C, as described previously [30]. After cell sheet detachment from the poly(*N*-isopropylacrylamide) (PNIPAAm)-grafted culture dishes, the sterilized hydrogel carriers were immediately placed on the apical surface of cell layers to create HA-CEC sheet constructs.

Due to its high regenerative capacity, rabbit corneal endothelium was treated with mitomycin-C (Sigma-Aldrich) to establish an animal model mimicking conditions of human corneas [30]. The rabbits were anesthetized intramuscularly with 2.5 mg/kg body weight of tiletamine hydrochloride/zolazepam hydrochloride mixture (Zoletil; Virbac, Carros, France) and 1 mg/kg body weight of xylazine hydrochloride (Rompun; Bayer, Leverkusen, Germany), as well as topically with two drops of 0.5% proparacaine hydrochloride ophthalmic solution (Alcaine; Alcon-Couvreur, Puurs, Belgium). In the operated eye of each rabbit, 0.1 mg/mL of mitomycin-C was injected into the anterior chamber. After treatment for 2 weeks to prevent CEC proliferation and migration, the cornea was penetrated near the limbus with a slit knife under the surgical microscope (Carl Zeiss), and the central 7 mm of corneal endothelium were scrapped gently with a silicone-tipped cannula without damaging Descemet’s membrane underneath.

The cell/biopolymer constructs were implanted in the anterior chamber through a 7.5-mm corneal/limbal incision (HFT and LFT groups, 6 rabbits for each group). The incision site was closed with 10-0 nylon sutures. Traumatized rabbit corneas receiving no transplantation served as control groups (*n* = 6). After surgery, 1% chlortetracycline hydrochloride ophthalmic ointment (Union, Taipei, Taiwan) was immediately applied to the ocular surface in all three groups. Each surgical eye received two drops of 0.3% gentamicin sulfate (Oasis) and one drop of 1% prednisolone acetate (Allergan, Westport, Co., Mayo, Ireland) four times a day during the follow-up period of 4 weeks. Ophthalmic evaluations were done after 4 weeks of transplantation. The corneal clarity was assessed using slit-lamp biomicroscopy (Topcon Optical, Tokyo, Japan). The ocular grading method used for biomicroscopic examinations is shown in Table 1. During clinical assessment, three parameters were recorded from rabbit eyes and were numerically graded on an increasing severity scale of 0–4. The means of the ocular scores for each parameter were quantitatively calculated to be the sum of the scores for each group, divided by the total number of eyes in that group. The total score was expressed as the summary of three mean ocular scores for each group. The CEC morphology and density in the rabbit eyes were observed and measured by specular microscopy (Topcon Optical). Each data point was an average of three independent observations.

**Table 1:** Ocular grading system used for biomicroscopic examinations.

Parameter	Ocular Score				
	0	1	2	3	4
Corneal edema	None	Mild	Moderate	Severe	N/A ^a^
Corneal cloudiness severity	Normal	Mild	Moderate	Severe	N/A ^a^
Corneal neovascularization	None	Mild	Moderate	Severe	N/A ^a^

^a^ Not applicable, because the biological responses are too severe to be observed.

### 3.7. Statistical Analyses

Results were expressed as the mean ± standard deviation (SD). Comparative studies of means were performed using one-way analysis of variance (ANOVA). Significance was accepted with *p* < 0.05.

## 4. Conclusions

Development of porous biomaterial carriers for cell sheet transplantation can potentially tackle the issues of fragility of tissue-engineered corneal endothelial monolayer. However, the effects of the cryogenic operation temperature-mediated pore microstructure of HA carriers on the material biocompatibility and cell delivery performance are yet to be investigated. As compared to their counterparts fabricated at 0 °C, the HA samples prepared at lower pre-freezing temperature may exhibit restricted porous structure, thereby further causing viability loss and functional abnormality of cultured CECs. In an animal model of corneal endothelial dysfunction, the wounded corneas receiving bioengineered CEC sheets and LFT carriers also show poor tissue reconstruction. The therapeutic efficacy can be improved by intraocular delivery of cell sheet grafts using HFT carriers. These findings suggest that the cryogenic operation temperature-mediated pore microstructure of HA carriers plays an important role in corneal endothelial tissue reconstruction.
